# Association syndrome de Williams et insuffisance surrénalienne

**DOI:** 10.11604/pamj.2017.27.10.8177

**Published:** 2017-05-05

**Authors:** Meryem Rchachi, Maazou Mahamane Larwanou, Hanan El Ouahabi, Farida Ajdi

**Affiliations:** 1 Service d’Endocrinologie, Diabétologie et Maladies Métaboliques, CHU Hassan II de Fès, Maroc; 2 Faculté de Médecine et de Pharmacie, Université Sidi Mohamed Ben Abdellah, Fès, Maroc

**Keywords:** Hybridation fluorescente in situ, syndrome de Williams, insuffisance surrénalienne, Fluorescent in situ hybridization, Williams syndrome, adrenal insufficiency

## Abstract

Les anomalies du développement décrites dans le syndrome de Williams, associent classiquement une dysmorphie, des malformations cardiovasculaires et un profil neuropsychologique particulier et d'autres troubles associés. Nous rapportons le cas d'une jeune fille âgée de 17 ans, issu d'un mariage non consanguin, chez qui le syndrome de Williams a été découvert suite à une exploration dans le cadre du bilan d'un retard staturo-pondéral. L'association a une insuffisance surrénalienne primitive en fait la singularité. La confirmation diagnostique avait nécessité une analyse cytogénétique et moléculaire. La prise en charge consistait à la mise en route de traitement pour l'insuffisance surrénalienne associé à une surveillance clinico-biologique.

## Abstract (en)

Williams syndrome is a developmental disorder including dysmorphia, cardiovascular malformations and a specific neuropsychological profile together with other associated disorders. We report the case of a 17-year old girl, born of a non-inbred marriage, with Williams syndrome discovered during an assessment of degree of failure to thrive. Its association with primary adrenal insufficiency makes it unique. Diagnosis is confirmed by cytogenetic and molecular analysis. Its management consists of the implementation of treatment for adrenal insufficiency associated with a clinico-biological monitoring.

## Introduction

Le syndrome de Williams et Beuren décrite en 1961, est un syndrome génétique rare qui correspond à une anomalie du développement associant classiquement une dysmorphie du visage assez spécifique, des malformations cardiovasculaires et un profil neuropsychologique particulier [1, 2]. Cette pathologie est généralement découverte suite au bilan malformatif dans le milieu cardio-pédiatrique. La confirmation du diagnostic quant à elle nécessite des analyses approfondies notamment la génétique moléculaire qui permet de découvrir l'anomalie chromosomique [3]. Les auteurs rapportent ici le cas d'une jeune patiente qui présente un syndrome Williams et Beuren découvert suite à un bilan d'un retard de croissance dont l'association a une insuffisance surrénalienne constitue son originalité.

## Patient et observation

**Figure 1:**
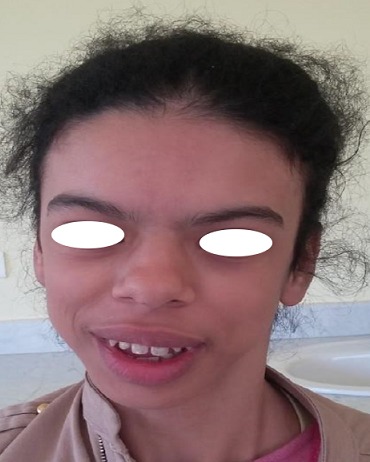
Le syndrome dysmorphique du syndrome de williams

Melle Z est une patiente âgée de 17 ans, issu d'un mariage non consanguin, admise pour exploration d'un retard staturo-pondéral. L'examen clinique à son admission notait un morphotype caractéristique du syndrome de Williams Beuren qui est fait d'un front bombé, crête du nez rectiligne avec extrémités bulbeuses, grande bouche avec lèvre inférieure large et éversée, dents écartés, épicanthus, strabisme et iris stellaire, un long cou et des épaules tombantes (Figure 1). Par ailleurs, La patiente présente une hyper sociabilité, une hypersensibilité aux sons extérieurs avec une préférence aux sons musicaux. On ne retrouve pas d'anomalies cardiaque ni phosphocalcique. Le diagnostic de certitude a été rapporté grâce à une hybridation in situ fluorescente qui objectivait selon les normes internationales le résultat suivant: (46,XX.ishdel (7)(q11.23) (ELN-)(3)), nucish ((ELNx1)(D7S522x2)(80)). Lors de son hospitalisation une insuffisance surrénalienne a été diagnostiquée et la patiente mise sous traitement substitutif à vie avec une surveillance clinique et biologique à la recherche d'une anomalie cardiovasculaire et ou des troubles métaboliques.

## Discussion

Le syndrome de Williams-Beuren est une entité clinique et génétique rare. Le syndrome clinique se traduit par une dysmorphie du visage assez spécifique, des malformations cardiovasculaires associées à un profil neuropsychologique particulier. Le diagnostic de certitude est facile à confirmer grâce à l'analyse cytogénétique notamment la technique d'hybridation fluorescente in situ (FISH) qui a connu un regain d'intérêt dans ce domaine. L'anomalie caractéristique est une micro délétion chromosomique en 7q11.23 [4, 5]. Plusieurs auteurs ont rapporté l'association de ce syndrome à d'autres désordres endocriniens dont les plus fréquemment rencontrés sont: l'hyper calciurie, l'hypercalcémie idiopathique, l'hypothyroïdie et la puberté précoce [6]. Nous rapportons le cas d'une jeune patiente qui présente un syndrome Williams associé à une insuffisance surrénalienne qui constitue à notre connaissance une des rares associations. Il s'agit d'une insuffisance surrénalienne qui a été découverte lors du dosage du cortisol plasmatique par le test au synacthène dans le cadre d'une exploration d'un retard staturo-pondéral. L'insuffisance surrénalienne au cours de ce syndrome est exceptionnelle et le mécanisme physiopathologique demeure méconnu.Le pronostic du syndrome de Williams-Beuren est dominé principalement par l'atteinte cardiaque [3], non retrouvée chez notre patiente nécessitant la mise en route d'une surveillance régulière.

## Conclusion

Le syndrome de Williams-Beuren est une maladie génétique rare qui s'associe souvent à des désordres endocriniens divers. L'association à une insuffisance surrénalienne est exceptionnelle, mais possible. Une exploration biologique de l'axe hypothalmo-hypophysaire-glandulaire doit être systématique afin de détecter certains désordres inhabituels comme le cas de notre patiente.

## Conflits d’intérêts

Les auteurs ne déclarent aucun conflit d'intérêt.
